# Using COM-B model in identifying facilitators, barriers and needs of community pharmacists in implementing weight management services in Malaysia: a qualitative study

**DOI:** 10.1186/s12913-022-08297-4

**Published:** 2022-07-19

**Authors:** Ali Qais Blebil, Pui San Saw, Juman Abdulelah Dujaili, K. C. Bhuvan, Ali Haider Mohammed, Ali Ahmed, Fatima Zohreine Houssenaly, Bassam Abdul Rasool Hassan, Anisha Kaur, Rohullah Roien

**Affiliations:** 1 School of Pharmacy, Monash University Malaysia, Bandar Sunway, Malaysia; 2 Department of Pharmacy, Al-Rafidain University College, Baghdad, 10001 Iraq; 3 Medical Research Centre, Kateb University, Kabul, 1004 Afghanistan

**Keywords:** Behaviour change, Community pharmacist, Malaysia, Qualitative study, Weight management

## Abstract

**Background:**

Previous qualitative studies exploring the experiences of community pharmacists (CP) in implementing weight management services (WMS) often lack a theoretical underpinning. This study applied the capability, opportunity, motivation, behaviour (COM-B) model to factors associated with WMS implementation among CPs to develop and recommend better intervention strategies.

**Methods:**

A qualitative study design was used by conducting in-depth, semi-structured interviews with CPs. All the interviews were audio-recorded and duly transcribed. The thematic analysis approach was used to analyse the data, and the themes generated were mapped onto COM-B model components.

**Results:**

The themes that emerged were (1) motivation of pharmacists and (2) knowledge and skills, which were identified as both barriers and facilitators, and (3) the barriers to implementation in aspects of social norms and resources. Factors were subsequently categorised into the subcomponents of the model: physical capability (e.g., training), psychological capability (e.g., lack of knowledge), physical opportunity (e.g., product range), social opportunity (e.g., stigma), automatic motivation (e.g., remuneration) and reflective motivation (e.g., CPs extended roles).

**Conclusions:**

In conclusion, programs or training For Cps should develop their psychological capability to change their behaviour by being more proactive in promoting and providing weight management services, with a vital educational component. This behavioural change will improve the promotion of this service and will help many customers who were unaware of this service. Learning opportunities will leave CPs to feel more empowered and overcome barriers to implementing and maintaining WMS in primary care. The study findings provided essential insights into the factors that affect this provided service in Malaysia. The results will help to encourage the embedding of nutrition counselling in academic curricula.

## Background

Obesity has become a leading health problem globally in many countries, especially in Asia [1]. It has become well known that obesity is one of the main modifiable risk factors for cardiovascular disease. According to the WHO criteria, the body mass index (BMI) classifies the overweight and obese with a BMI of 25-29.9 kg/m2 and >30 kg/m2, respectively [2]. Several reports have shown that numerous non-communicable diseases such as dyslipidaemia, type II diabetes mellitus, cardiovascular disease, hypertension and certain types of cancer were related to overweight or obesity, further enhancing the burden of diseases and the mortality rate [3–5]. In Malaysia, the prevalence of overweight and obesity was similarly observed in an increasing trend among adults aged 18 years and older [6]. Malaysia is now ranked as the highest in Asian countries according to the World Health Organisation (WHO) reports, with the percentages of overweight or obese males and females being 64% and 65%, respectively [7].

In the past decade, various strategies and efforts have been implemented in Malaysia to combat the high prevalence of obesity. Obesity remains a national priority in Malaysia [8]. In 2014, the Institute for Public Health conducted a dialogue on obesity research to discuss the magnitude of the obesity problem and categories of obesity research undertaken by various institutions, including the Ministry of Health, the National Institutes of Health and local universities [9]. In the latest report of Nutrition Research Priorities in Malaysia (NRPM) (2016-2025), overweight and obesity research priority area was identified with the focus on improving understanding of the epidemiology of obesity, management of obesity, the effectiveness of the intervention and developing new modalities [10, 11].

Community pharmacies are strategic and important locations for opportunistic screening due to pharmacist availability and the skill to implement various health and medication management services. It is estimated that most of the population in Malaysia frequent community pharmacies for health advice and purchase (over-the-counter) OTC products [12, 13]. It has been reported that these pharmacies have been known to provide various health-related services such as cardiovascular diseases, asthma, and smoking cessation [14]. Thus, community pharmacists (CPs) are best positioned also to offer weight management services (WMS). Through WMS, pharmacists can meet with clients during their regular visits to the pharmacy to provide motivational counselling to ensure efforts are followed up in improving their diet, increasing their physical activity and achieving their weight loss goals in a healthy, sustainable way. As medicines experts, pharmacists can also provide information regarding weight-loss medicines or other weight loss products found in pharmacies. A scoping review of nine studies concluded that pharmacist-led WMS could have a positive potential effect on weight-loss management [15]. A study was conducted to assess the success rate of pharmacy services in supporting obese individuals during weight loss, and there were two assessment points (3 and 6 months) [16]. At three months, the remaining participants' mean weight reduction (110/281 subjects) was -3.0 kg. While at six months, the study reported an additional reduction from baseline with a mean change of more than -4 kg of the remained participants (59/281 subjects) [16]. A systematic review showed that the weight management service could produce reasonable weight loss at 12 months between 1.1 – 4.1 kg [17]. The study reported health benefits of clinically significant weight loss, which happen with a loss of ≥ 5% baseline weight. These benefits include improved lipid profiles, improved glycaemic control, reduced blood pressure, and reduced osteoarthritis-related disability [17]. Despite its benefits, few studies have reported that the customers viewed CPs as ‘medicine experts’ rather than wellness experts, thus may not appear suited and knowledgeable to discussing about healthy lifestyle issues [18–20]. Moreover, a lack of patient expectations was identified as one of the main barriers to providing relevant services to obese patients [20].

Often, lack of program monitoring and evaluation as highlighted in the National Plan of Action for Nutrition in Malaysia, prompted for data to be used more effectively to analyse bottlenecks and gaps, which can then be addressed to enhance program implementation. While pharmacist-led WMS has been studied in Malaysia [21], self-administered questionnaire investigating the knowledge, attitudes, and perception of CPs does not provide in-depth insights into the opinions and experiences of the participants to show how WMS might best work and find potential barriers to help alter professional behaviour.

**Fig. 1:**
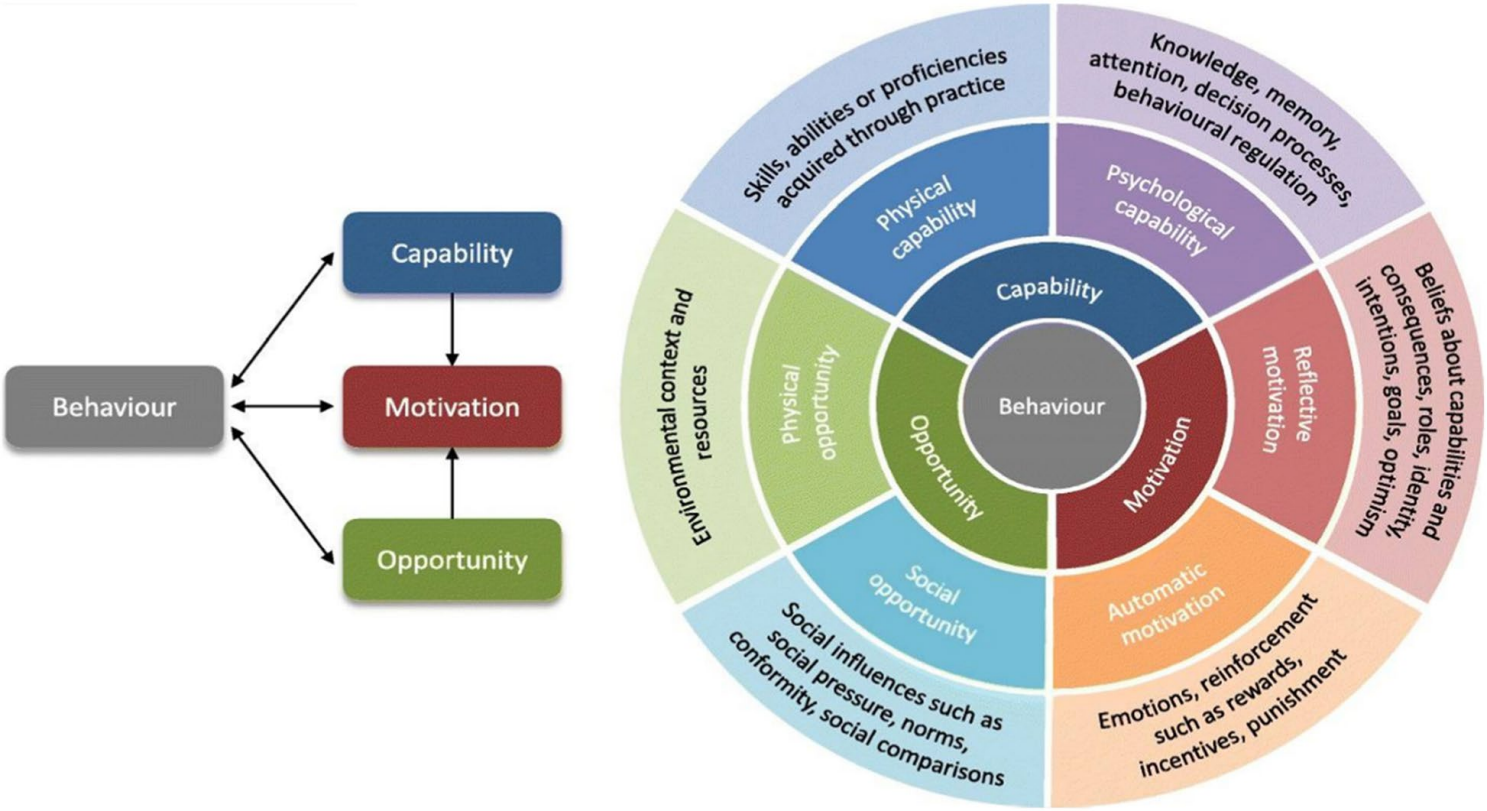
The COM-B model [22]

Practice change requires behaviour change in several stakeholders supported by a theoretical understanding of behaviour [22]. Michie et al. [23] developed the Capability, Opportunity, and Motivation Behaviour (COM-B) model, a comprehensive model, to guide understanding of the behaviour of interest and identify behaviour targets as the basis for the design of interventions. COM-B postulates that for one to engage in a particular behaviour, they must have the capability physically and psychologically, (and have the social and physical opportunity to perform the behaviour, and in addition, be motivated in both automatic processes (habit and impulses) and reflective processes (intention and choice) (Fig. 1).

In the COM-B model, additional potential influences on behaviour can be considered from the outlined explanatory components, making this a suitable model for this study. As such, if the desired behaviour is not occurring, then an analysis of the determinants of the behaviour will help define which components need to shift for the desired behaviour to occur. COM-B lies at the centre of the Behaviour Change Wheel, commonly used as starting point of intervention development [23]. Mapping the COM-B components facilitates the selection of intervention strategies that are likely to be appropriate and effective in addressing the facilitators and barriers for each component. This model can contribute insights into improving CPs’ uptake in WMS [23].

Another qualitative study exploring the experiences of CPs in implementing WMS lacked a theoretical underpinning [24]. To the authors' knowledge, based on searching for original articles via searching the database such as Scopus, Ovid Medline and google scholar, this model has not been employed to study WMS among CPs, though it has been effectively applied in many other health behaviours [25–27]. Several dietary and weight management studies found that COM-B has been useful in identifying barriers and facilitators that represent potential targets for intervention design [25–27]. Grounded in behaviour change theory, the usage of the COM-B model in this study will assist in building a coherent framework to suggest future areas for implementation and increase uptake of WMS among CPs. This study aimed to explore facilitators, barriers, and needs of community pharmacists in implementing weight management services in Malaysia using the COM-B model. In addition, a socioecological approach that explores multiple-level factors between individuals, social, physical and policy environment was taken to understand better where to apply the intervention facets [28].

## Methods

### Study design

Given the exploratory nature of the research question, qualitative methodology was applied to enable the collection of in-depth information to understand and interpret the personal experiences of CPs in their interactions with clients in their daily practice. We applied the phenomenological method by requesting community pharmacists (the participants) to describe, understand and interpret the meanings of experiences of providing the weight management services. In-depth individual interviews were conducted with CPs from different socioeconomic areas in Klang Valley, Malaysia. Qualitative data was collected using a phenomenological approach to explore and explain the common understanding of CPs’ experiences in offering WMS while preserving their opinions' richness, breadth, and depth.

### Sampling and recruitment

This study was carried out between June and July 2019. This study was conducted in Selangor and Kuala Lumpur with fully registered CPs with the Pharmacy Board of Malaysia, either owning or working in a chain or independent pharmacies and irrespective of WMS implementation at their practice sites. A purposive sampling strategy was used to achieve maximal variation among the cohort. In this study, the inclusion criteria were full-time community pharmacists with a minimum experience of 1 year. Community pharmacists who were appointed pharmacy managers in their respective outlets were also recruited to understand their perspectives on the management and operational aspects. In addition, the participants need to be able to converse in English.

We recruited participants through posting online via Facebook, LinkedIn and through personal communication of the researchers. We screened participants for eligibility, excluding those not practising or practising in other settings, such as hospitals, nursing homes, or pharmaceutical companies. There were no incentives provided for participation in the study. Interviews were conducted until data saturation was achieved.

### Data collection

The items of the data collection form were developed based on an in-depth literature review, expert opinion and conceptual framework [29, 30]. The data collection form, which was a semi-structured interview guide, consisted of two parts. The first part consisted of 12 closed-ended items that explored the weight management practices in community pharmacies, including the provision of lifestyle advice, orlistat dispensing, complementary and herbal medicine, fat burners, the number of times per week the pharmacists dispensed both prescriptions and OTC weight-loss products and medicines, using the five options from “none” to “10 or more”. The second part included 12 open-ended questions that focused on the service and the weight management product provided by the pharmacists and their motivation to provide it. Besides, the participants were asked if they received any educational training for weight management services and evaluated their interaction with customers looking for weight management products and services. Furthermore, a demographic information section was also included.

Prior to data collection, the interview guide was undertaken for content and face validation. The interviews were piloted on two experienced researchers in qualitative studies at Monash University Malaysia and four CPs using a combination of argumentative and cumulative techniques [30] to ensure relevance and importance of the questions in the interview guide and that participants understand the vocabulary used throughout the interview.

The interviews were conducted face-to-face by a trained qualitative researcher (FH). FH received training in conducting qualitative research during the academic year 2019 in Malaysia. All the interviews were conducted in respective community pharmacies for confidentiality and to avoid distraction. After a brief introduction of the purpose of the study, the interviewer obtained oral consent from the interviewee to start the recording and began the semi-structured interview according to the agreed guide. In addition, the interviews were audio-recorded to allow for accurate transcription prior to analysis. The qualitative and quantitative data collection occurs in *parallel,* and analysis for integration begins well after the data collection process has been completed.

### Data analysis

Anonymised interview transcripts were transcribed verbatim and imported into NVivo (QSR International, version 12). A seven-step approach to thematic analysis was performed: data familiarisation, coding of the dataset (nodes), identification of nodes, review and revision of nodes, interpretation of patterns across nodes and generation of emerging themes and subthemes. The two researchers (AQB and PSS) independently coded the data set and then compared their coding. At each stage of the process, discussion among team members resolved any discrepancies to produce an analysis of what would help or hinder the implementation of WMS among CPs. The results presented in the form of themes were described using illustrative quotes from each participant, who is assigned a consecutive code (i.e. participant 1 = P1). The identified themes were then classified into the coding framework which comprised of 3 main elements and six subcomponents (Fig. 1). The mapped themes into the COM-B model were identified at the patient, provider and system-level to reflect the structure of the socioecological model, identifying areas from the microlevel to macrolevel which need to be targeted to create and sustain behaviour change. Using the quantitative data sets to understand the participants’ practices relating to WMS, the qualitative findings were integrated to triangulate input about their needs, ways to address barriers and ways to implement changes.

## Results

A total of 25 participants from independent (44%) and chain community pharmacies (56%) were interviewed (Table 1). The mean working experience among the participants was 5.72 ± 4.89 (1 - 16 years). The participants work an average of 48.12 ± 6.16 hours per week (24 - 60 hours) in the community pharmacy, where the majority spent most of their time in customer support (Table 2). It was also noted that more than 80% of participants had not received any form of formal training with limited access to educational resources on weight management. Despite that, about half of the participants have provided their customers an average of 2 to 5 products and weekly consultations relating to weight management .

**Table 1:** Participants demographic background

Characteristics	*N* =25	Percentage
Gender		
Male	9	36%
Female	16	64%
Age		
under 25	1	4%
25 – 29	13	52%
30 – 39	9	36%
50 – 59	1	4%
60 and above	1	4%
Ethnicity		
Chinese	22	88%
Indian	1	4%
Malay	2	8%
Job description		
Employee pharmacist	12	48%
Pharmacy manager	12	48%
Pharmacy owner and manager	1	4%
Type of community pharmacy		
Multi-outlet independent pharmacy	9	36%
Single-outlet independent pharmacy	2	8%
Chain pharmacy	14	56%
Years of practice as a pharmacist		
5 years or less	16	64%
6 – 10 years	6	24%
11 – 15 years	1	4%
16 – 20 years	1	4%
More than 20 years	1	4%
Education qualification		
Master’s Degree	5	20%
Bachelor’s Degree	20	80%

**Table 2:** Practice characteristics of study participants

Items	*N* = 25	Percentage
Percentage of time (from daily working hours) spent attending to customers		
0 – 25%	1	4%
26 – 50%	4	16%
51 – 75%	5	20%
76 – 100%	15	60%
Training support in weight management in the last 12 months		
No	21	84%
Yes	4	16%
Average number of over-the-counter products recommended (per week) for weight management in the last 3 months		
None	2	8%
Less than 2	9	36%
2-5	13	52%
6-10	0	0%
More than 10	1	4%
Average number of consultations (per week) on weight management in the last 3 months		
None	2	8%
Less than 2	9	36%
2-5	12	48%
More than 5	2	8%

### Key themes identified and mapped onto the COM-B model

Results of the mapping exercise (Table 3) highlighted main themes that revolved around the pharmacists’ motivation in promoting WMS, knowledge and skills for pharmacist to carry out WMS and the barriers to WMS implementation in aspects of social norms and resources. The main driver for service provision stemmed from the positive intentions among CPs in improving community health outcomes while seeking training opportunities to be able to carry the role effectively. These factors were subsequently categorised into the subcomponents of the model: physical capability (e.g., training), psychological capability (e.g., insufficient knowledge), physical opportunity (e.g., product range), social opportunity (e.g., stigma), automatic motivation (e.g., remuneration) and reflective motivation (e.g., CPs’ extended roles).

**Table 3:** Overview of how themes of barriers and facilitators in promoting WMS fit within the COM-B and socioecological domains

COM-B	Framework	Subthemes	Concepts	Socioecological domain
Capability	Physical	Training	No formal training, awareness	Provider-level
			Formal training, company, manager	System-level
		Routine lifestyle counselling	Lifestyle modification, diet, exercise, BMI	Provider-level
	Psychological	Expectations of fast-acting weight loss	Impatience, request for fast and effective products	Patient-level
		Self-obtained information	Sales representatives, self-reading, online or printed materials	Provider-level
		Insufficient knowledge	Standard approach, plan	Provider level
Opportunity	Physical	Lack of guidelines	Unaware of procedures, professional integrity	Provider-level
		Practicality and useful guidelines	Current practice, individuality	System-level
		Wide product range	Approach to WMS, lifestyle intervention	Provider-level
	Social	Service demand	Aesthetic, female, comorbidities, overweight	Patient-level
		Stigma	Culture, rude, insensitive	Provider-level
Motivation	Reflective	CPs’ extended role	History-taking, avoid drug-herb interactions	Provider-level
		Liaising with other health providers	Nutritionist, doctor, team platform, referral	System-level
	Automatic	Remuneration	Unwilling to pay for service, uncertified	Patient-level
			Not for profit, free of charge, consultation	Provider-level
		Business aspect	Drive pharmacy sales	Provider-level
		General health improvement	Lifestyle management, health	Provider-level

### Capability

(i) **Physical capability**

The training was extensively discussed among the participants and claimed to be essential to develop the confidence and skills in delivering WMS. As evidenced in both the quantitative and qualitative findings, more than 80% of the participants revealed that they had no formal training in WMS in the last 12 months. They were also mainly from the pharmacist group with working experience below five years. One participant who received formal training from the pharmacy headquarters had WMS as part of the company initiatives. Participants welcomed more training in step-by-step procedures and clinical conditions.

*“If I have the training, I can (conduct weight management services) actually… maybe there is some guidance on proper steps like what I should start first… what am I supposed to look for…” (P23)*

The majority of the respondents claimed that they do not provide WMS but provide counselling on lifestyle modification, especially diet and exercise intervention to reduce weight. Participants, who were prompted to elaborate on WMS, named the calculation of body mass index (BMI) as a common measure.

*“Counselling on the product yes… but weight management itself not yet. But of course, we do tell them how to lose weight and what is their target weight and talk about BMI”* (P25)

All participants showed a positive attitude towards a role in providing weight management products and services in Malaysia, with conditions that more training and awareness campaigns should be held to improve their knowledge and skills.

*“Pharmacist role in weight management is very important because we are the front liners and first healthcare provider that the patient can approach before going to the doctor, plus we are providing free consultation because at the doctor they have to pay. We definitely need more training on weight management and more tools, like BMI machine or the machine that test the layer of fats in our body.”* (P25)

(ii) **Psychological capability**

Participants claimed that clients often requested products that would work fast and effectively. Some participants made initiatives to redirect expectations and instil awareness of healthy weight managing interventions to address this misconception.

*"They want something fast and effective, which they can see results in less than a month… some wants result in weeks. Normally that is their expectation. But we educate them on the expectation… If (you try to reduce weight) too fast, it is not healthy."* (P8)

The participants revealed that they usually received more information through talks from product representatives. Some participants have resorted to self-reading through online materials and health magazines. Participants also admitted that their own experiences influenced how they delivered advice.

*“What I did (study on my own) was more on nutrition… what to eat, what not to eat… I just do it myself; I just pay and go and attend classes on my own”* (P7)

Some pharmacists, however, claimed that they did not have a standard approach to weight management and provided advice on lifestyle intervention and product recommendations, as they would with other requests received in a community pharmacy setting.

*“In general, we just provide advice in response to their request, so we don’t have a formulated plan.”* (P20)

**Opportunity**

(i) **Physical opportunity**

The majority of participants stated that they were not aware of any guidelines on weight management procedures other than maintaining professional integrity and ethical responsibilities while providing the service to clients.

*“Not that I know of (in terms of guidelines) … I would just say try our best using our professional knowledge to tell whatever that we know to help the patient…”* (P3)

On questioning the utilisation of clinical practice guidelines for weight management in Malaysia, the participants felt that the guidelines were not as helpful as they are not customised to the current practice.

*"We don't really use it because sometimes it is not really practical… the service should be customised according to every individual because everyone practices a different lifestyle."* (P19)

Two participants described a wide range of WMS provided, including weight measurement, body fat analysis, calculation of body mass index, advice on healthy eating, and providing weight loss products. For the rest of the participants, preferences on approach to weight management differed. Half of the participant group opted for lifestyle and dietary modification as the first choice of recommendation for their clients. Two participants stressed that the common root of health issues was inappropriate lifestyle and diet; therefore, changes to these factors should be implemented to sustain long-lasting health.

*“It is always the lifestyle problems (that causes these health problems) … either they sleep late, (feeling) stressed, not eating well, not eating veggie at all, drinking too much boba tea.”* (P7)

*“Lifestyle first then products, because if they only take it (weight loss products) for a week then it will be useless so normally I would ask them to do the diet (interventions) first.”* (P25)

Another half of the participants preferred to recommend products complemented with advice on lifestyle management. Products supplied are either in the form of medicine (i.e., orlistat) or supplements mostly for detox or fat burning (e.g., garcinia cambogia). When further questioned their rationale for such recommendation, two participants revealed that the main reason is the perceived lack of motivation on lifestyle modification (such as exercise) among the clients.

*“Go for the products first because the lifestyle is very hard to change.”* (P24)

*“I don't really ask about exercise because I just assumed, they don't really do much exercise… if they (have) come to a retail store and ask for products then (that means) they want to take a product.”* (P20)

(ii) **Social opportunity**

WMS in community pharmacies was largely opportunistic and reactive in nature (i.e. at the request of individual clients). Some participants also felt that clients were not comfortable discussing about weight-related issues with pharmacists. On the other hand, participants felt that initiating discussions about weight management can be also challenging and deemed as being culturally rude and insensitive.

*“No, I think in a lot of culture weight is a sensitive issue, so you don’t suggest this kind of thing unless they ask you.”* (P17)

Overall, the participants revealed two main groups of clients who would seek WMS in community pharmacy: (1) clients with chronic diseases including diabetes, hypertension and hypercholesterolemia, and (2) middle-aged women.

*“Normally (those who come are) like obese… and then we do get people with (high) cholesterol (levels) and then they want to lose weight so that they can reduce their cholesterol level.”* (P8)

However, they described that women clients who were more interested in asking for information on weight management do not have issues with their weight. In fact, the participants felt that these clients were self-conscious about losing weight for aesthetic reasons. On the contrary, the participants claimed that the overweight clients were often the ones who seldom bring up the topic of weight management.

*“I realised that those overweight, obese customers, are not the ones that ask questions about weight management. (Question are from) those who are very aware and very conscious about their weight… yes”* (P25)

*"The people that would come would be definitely like younger girls or women in their 30s - 40s… they have already gone to a spa and everything regarding their beauty… they don't really have a weight problem that much."* (P7)

Two participants added that men clients were oblivious about their weight, even though they felt that men were generally more likely to be overweight.

*"People with a weight problem are more… (likely) men, and they are very stubborn who don't want to control their diet… so they don't really care about their weight."* (P7)

A few participants would take the advantage to highlight health problems associated with obesity and the benefits of weight reduction in managing chronic diseases to initiate conversations with their clients.

*“First, I will try to relate their disease with obesity… they have any vascular diseases, joint pain… then just let them know that they have to really take care (in managing) obesity because it will indirectly affect the current diseases”* (P5)

Therefore, the majority of the participants shared that identifying and approaching potential clients for weight management service was usually initiated through a background check to first understand the health status of their clients.

*“The general process… firstly I will get their weight and their height first to see whether it is obese or just overweight. Then, I will ask them what is their lifestyle like... what do they eat, how is their lifestyle, is it sedentary or an active lifestyle… so (I want to) get to know their background first before introducing anything to them. That is my procedure.”* (P11)

**Motivation**

(i) **Reflective motivation**

All participants claimed that more thorough history-taking was done mainly for clients with chronic diseases, such as medications and health conditions, to avoid drug interactions with a recommended product.

*“Of course we need to ask whether they have any concomitant disease and also is there any medication they are taking… let's say the product that you want to recommend… whether there is any interaction… whether it will affect the absorption of the other medicine.” (P14)*

Some participants also saw the opportunity of offering WMS as a platform to liaise with other healthcare providers. Referral to the doctor’s office was also routinely done by most participants.

*"Nutritionist, pharmacist and doctor (can work) together to advise… a nutritionist would suggest meal plans, for us (pharmacists) will be advising on lifestyle and supplements, doctors (also) pharmacists will also weigh the risk for other comorbidities."* (P3)

*If they haven't done a check-up (at the doctor's office), let's say for the really obese patient, usually, we will advise them to go for a check-up especially cholesterol."* (P14)

(ii) **Automatic motivation**

When exploring whether WMS in community pharmacies should be charged, participants mentioned that inquiries and support were infrequent for profits to be churned out. Only two participants charged MYR50 for the weight management service that was provided. Other participants revealed two main reasons for offering weight management advice free of charge were: (1) they were not formally trained to provide such service, and (2) perceived that clients were unwilling to pay for the provision of advice.

*“(Charge on) consultation? No. Because to me in a retail pharmacy or community pharmacy, it is my job to provide guidance towards the patients on how they should manage their weight, so I don’t see any point on charging them unless of course if they buy a product from my shop.”* (P12)

*“First of all, we are not certified for weight management consultation… we are not professionals in that field, and there is no specific (training) program for it... for people to actually come here on a weekly basis, I don’t think people would want to pay for it because you wouldn’t pay for advice though, you can just get it from google.”* (P2)

While a few participants acknowledged that WMS could also help drive sales of the pharmacy, all participants unanimously agreed that the primary motivation was really to help improve the health of individuals since such services were usually offered free of charge.

*“To improve their lifestyle management and get them to be better controlled in their health… Motivation mostly for better health.”* (P15)

*“Not really (for business), it’s more towards for patient’s health.”* (P12)

## Discussion

This mapping of the themes onto each COM-B component demonstrated considerable improvement gaps. Both facilitators and barriers were tightly related to the education and awareness of WMS in both pharmacists and consumers. This is unsurprising as education level had been demonstrated to be highly associated with health beliefs [31, 32]. At the patient level, findings in this study showed that consumers who expected quick-fix products to manage weight might indicate limited awareness and knowledge of weight management. Other studies have also shown that poor knowledge and misconceptions about weight management were prevalent among consumers in Malaysia [21, 33, 34]. So far, only a sub-population of consumers were perceived to utilise WMS in a community pharmacy. The participants felt that pharmacists were not favoured as sources of advice on weight management. Reasons may be due to the lack of public awareness about WMS [21] and pharmacists' own perceived inability to provide useful advice and their limited knowledge about WMS and physical activity [21, 35, 36].

This study found that there is infrequent counselling for overweight and obese patients and such was also reported among healthcare providers in other studies relating to weight management [37–40]. Since CPs are easily accessible, they play vital roles in preventing public health hazards from obesity. This highlights the potential to promote community pharmacy services as patients are commonly unaware of what the pharmacies could offer. There is also a common consensus that their perceived status as “shopkeepers” would not change unless pharmacists took the initiative [41]. Pharmacists can also gradually transform the image of their premises into healthy living centres and repurpose retail space into consultation rooms or healthy lifestyle information areas. Given the skills and environment for professional consultations, CPs should even be more motivated to expand WMS [35, 42]. Studies have also shown that consumers were generally in favour of CPs providing WMS, which can be an added advantage to implementing such a service [35, 43]. Thus, the pharmacists will need to work harder to transform the patients' perceptions to be considered trusted clinical advisers. This affirmative perception positively will impact patients’ willingness to participate in pharmacist-provided WMS. Patients themselves will also need to put in effort in combating obesity issues. The perceived lack of patients’ responsibility to own health [37, 38, 44] led to several strategies proposed to alleviate social bias, with information-based approaches being the most common intervention [45–48].

Unlike their hospital counterparts where training pathways are linked to expected career progression, CPs in Malaysia do not generally have a clear training programme. In fact, participants in this study attributed their lack of knowledge and confidence in WMS due to limited access to training opportunities in carrying out the role. This is also observed in varying interpretations from study participants about the roles and activities that is offered in community pharmacist-led WMS. In England, for example, most providers participate in a training course prior to service delivery. The training and refresher courses covered multiple points, such as approaching and motivating clients and educating them on nutrition, exercise, and obesity [49]. CPs should also be open to being trained by other health professionals, such as dieticians, who are skilled in weight management and behaviour change strategies [50]. Pharmacy managers should invest in training programs for CPs, which is crucial to the business in the long run. Pharmacy organisations and local health departments can actively introduce various training for community pharmacists. With increasing education and training on weight management, pharmacists’ knowledge and confidence can significantly be increased [51–53]. Customers will also remain loyal if they are being served and advised by knowledgeable pharmacy staff.

In Malaysia, although the evolution of academic curricula has progressed to match the shift in the pharmacy profession towards a more patient-centred delivery of healthcare services, more focus on WMS is still needed. Nutrition and lifestyle counselling can be embedded in pharmacy education in universities to enhance foundational knowledge in health and wellness [54, 55]. In Australia, the weight management skills workshop incorporated in pharmacy curricula included case-based learning, hands-on experience, role-play and group discussion [56]. This would make an interesting interprofessional education topic a pedagogical approach for pharmacy and dietician students to learn about, from, and with each to enable effective collaboration in the community weight management program. In existent practice, WMS is a platform for a multidisciplinary team effort in primary care [42, 57].

Guidelines remained as an essential facilitator, as highlighted by the participants in this study. Several countries have developed tailored guidelines for WMS focused on obesity. For example, American Guideline for the Management of Overweight and Obesity in Adults 2013 [58], NICE UK guidelines on prevention [59] identification, assessment, management [60], and lifestyle services [61] provide evidence-based recommendations on cardiovascular risk and complications of overweight/obesity, clinical assessment, recommendations for target goals, diet, physical activities, behavioural and psychological therapy, lifestyle, pharmacological and surgical interventions as well as treatment for comorbidities. A blanket approach may, however, risk not capturing specific population groups. Mercer critically appraised the usefulness of guidelines in the management of obesity by illustrating how UK NICE guideline, one of the most comprehensive guidelines ever published on obesity, fit in the clinical practice setting [62]. Therefore, guidelines are best amalgamated to current local practice where people can be assessed for suitability for different weight management interventions.

In this study, participants provided free of charge services such as weight measurement, BMI calculation and advice on weight management, even though remuneration is needed to ensure the sustainability of community pharmacy services. However, there is generally low patients’ willingness to pay an additional cost for services delivered by community pharmacies [12, 35, 63, 64]. One possible reason is that consumers feel uncomfortable paying out-of-pocket for services usually offered free of charge. Reimbursement models observed in other nations such as Scotland, Denmark, and the United Kingdom take many forms, using referral points to introduce fee-for-service. These innovative models include payment of a single commitment fee [50, 65, 66], reimbursement for every patient attending appointments at the pharmacy [50], per consultation session [50, 67, 68] or payments for provision of replacement staff while another staff was trained for the program [50]. Another consideration to investigate would be a cost-evaluation of WMS and workload implications before reimbursement to CPs can be realised.

The subthemes mapped onto the COM-B model mirrors the hypothesised relationships between components of the model; where behaviour an interplay between capability, opportunity and motivation. For example, pharmacists who lacked training in WMS (physical capability) and knowledge about weight management (psychological capability) were less confident in providing advice and carrying out their role in weight management (reflective motivation). A wider approach is also required, intervening at the system-level. The level of change and capacity building required in community pharmacy is complex and will require significant organisational support to occur.

As with qualitative findings, our study is limited in that the sample was confined to community pharmacists in the urban areas of Malaysia. Accordingly, further future studies with large scale sample size are needed to generalise the capture the real picture of the current situation of providing this service in overall Malaysia. Another possible limitation is response bias that participants may have given socially desirable responses to present the best version of themselves. Although findings were community pharmacists’ perceptions of the situation, which may not present the holistic picture of WMS, they nevertheless inform opportunities to encourage service implementation. Future further research is needed for exploring public’s perception and opinion on the role of pharmacists in WMS and their acceptance towards such services in community pharmacies.

## Conclusions

In summary, CPs’ physical and psychological capability should be developed to change their behaviour, with a strong educational component. This important gaps can be supported at the system level for strategic plans and policies to be implemented accordingly to ensure relevant and continuous training is carried out in correspondence to customised rules and guidelines. Providing learning opportunities will leave CPs to feel more empowered and overcome barriers to implementing and maintaining WMS in primary care.

## Data Availability

The datasets generated and/or analysed during the current study are not publicly available as it contains personal information of the participants. However, the information was blinded before data analyses. The data are available from the corresponding author on reasonable request.
